# Short term feeding of industrial hemp with a high cannabidiolic acid (CBDA) content increases lying behavior and reduces biomarkers of stress and inflammation in Holstein steers

**DOI:** 10.1038/s41598-022-07795-z

**Published:** 2022-03-07

**Authors:** Michael D. Kleinhenz, Mikaela Weeder, Shawnee Montgomery, Miriam Martin, Andrew Curtis, Geraldine Magnin, Zhoumeng Lin, Jason Griffin, Johann F. Coetzee

**Affiliations:** 1 Department of Clinical Sciences, College of Veterinary Medicine, Kansas State University, 1800 Denison Ave., Manhattan, KS 66502 USA; 2 Department of Anatomy & Physiology, College of Veterinary Medicine, Kansas State University, 1800 Denison Ave., Manhattan, KS 66502 USA; 3 Institute of Computational Comparative Medicine (ICCM), Kansas State University, 1800 Denison Ave., Manhattan, KS 66502 USA; 4 John C. Pair Horticulture Center, Kansas State University, 1901 East 95th St South, Haysville, KS 67060 USA; 5 Present Address: Department of Environmental and Global Health, College of Public Health and Health Professions, University of Florida, Gainesville, FL 32610 USA

**Keywords:** Pharmacology, Preclinical research

## Abstract

Industrial hemp (IH) is defined as *Cannabis sativa* containing < 0.3% delta-9 tetrahydrocannabinol (THC) and was legalized in the 2018 Farm Bill. The impact of cannabinoids in IH fed to livestock, especially after repeat exposure, has not been thoroughly investigated. Sixteen male castrated Holstein cattle weighting (± SD) 447 ± 68 kg were enrolled onto the study. Cattle were allocated into two treatment groups either receiving IH (HEMP, n = 8) or a control (CNTL, n = 8). Cattle in the HEMP group were fed 25 g IH mixed in 200 g of grain once a day for 14 days to target a daily dose of 5.5 mg/kg of cannabidiolic acid (CBDA). Behavior was continuously monitored with accelerometers and blood samples were collected at predetermined time points for plasma cannabinoid, serum cortisol, serum haptoglobin, liver enzymes, serum amyloid A, and prostaglandin E_2_ concentrations. The HEMP group spent a mean 14.1 h/d (95% CI 13.6–14.6 h/d) lying compared to the 13.4 h/d (95% CI 12.9–13.8 h/d) for the CNTL cattle (*P* = 0.03). Cortisol concentrations in the HEMP group were lower than the CNTL group (P = 0.001). Cattle in the HEMP group demonstrated an 8.8% reduction in prostaglandin E_2_ concentrations from baseline compared to a 10.2% increase from baseline observed in the CNTL group. No differences for haptoglobin or serum amyloid A were observed. These results suggest that feeding IH with a high CBDA content for 14 days increases lying behavior and decreases biomarkers of stress and inflammation in cattle.

## Introduction

With the legalization of industrial hemp [*Cannabis sativa* containing < 0.3%delta-9 tetrahydrocannabinol (THC)] in the 2018 United States Farm Bill, interest in IH has grown. There are numerous uses for IH and its various plant components. These uses include human therapeutics, fiber for plastics and hempcrete, and biofuel production^1–3^. Additionally, there is interest and potential for the inclusion of IH and IH by-products in animal feeds. The nutritional content, digestibility, and cannabinoid concentrations of various IH plant components has recently been described^4^. These data show that certain IH plant components may be suitable for inclusion into cattle rations since they have favorable crude protein and digestibility profiles.

Recently, we demonstrated that cannabidiolic acid (CBDA) is readily absorbed from the rumen in cattle following a single dose of IH flowers cultivated for cannabidiol (CBD) oil production^5^. However, there are no published studies describing the effects of cannabinoids on cattle health and behavior. If hemp is to be utilized as an ingredient in the ration of cattle, knowing the pharmacokinetics and potential biological effects of cattle exposed to repeated doses of IH is prudent. These requisite data are needed if IH and IH by-products are to be considered by the US Food and Drug Administration (FDA) and the Association of American Feed Control Officials (AAFCO). The objectives of this study were to determine the: 1) plasma concentrations and pharmacokinetics of cannabinoids during a 14-day feeding period; and 2) impacts of feeding IH on cattle activity and blood inflammatory and stress biomarkers.

## Results

### Plasma cannabinoid concentrations and pharmacokinetics

**Figure 1:**
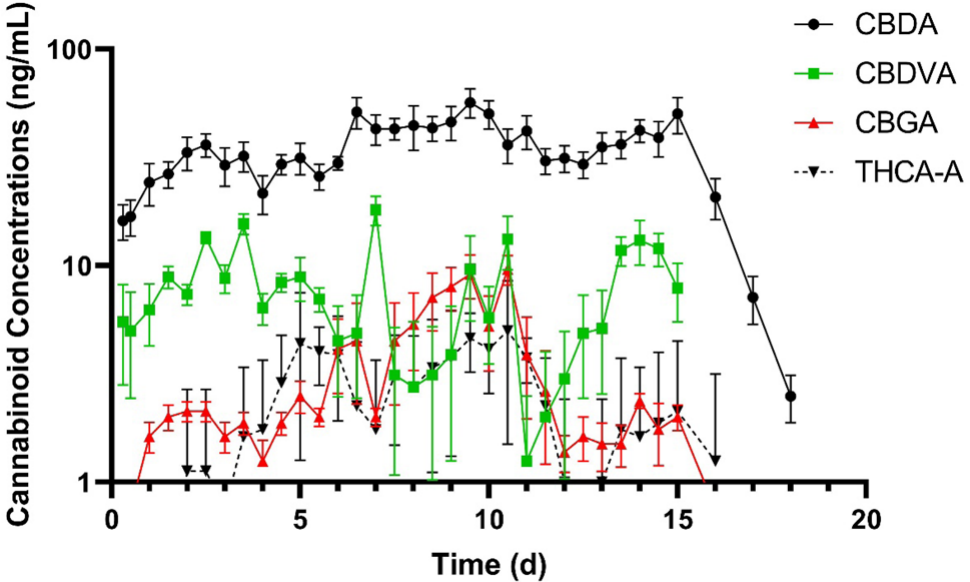
Mean plasma concentrations (ng/mL) of cannabidiolic acid (CBDA) at 5.5 mg/kg, cannabidivarinic acid (CBDVA) at 0.02 mg/kg, cannabigerolic acid (CBGA) at 0.2 mg/kg, and tetrahydrocannabinolic acid-A (THCA-A) at 0.1 mg/kg, over time in cattle feed industrial hemp once a day for 14 days (n = 8).

No cannabinoids were detected in any of the CNTL cattle samples. Of the 16 cannabinoids included in the analysis, 10 were below their level of detection at all time points. Cannabinoids detected were cannabidiolic acid (CBDA), cannabidivarinic acid (CBDVA), cannabidiol (CBD), cannabichromenic acid (CBCA), cannabigerolic acid (CBGA), and tetrahydrocannabinolic acid A (THCA-A). Plasma CBDA concentrations over time are shown in Fig. 1. Final cannabinoid doses on a milligram per kilogram basis are listed in Table 1. The pharmacokinetic parameters of CBDA for all HEMP cattle are summarized in Table 2. In the initial IH feeding period, the geometric mean maximum concentration of CBDA was 22.1 ng/mL and the geometric mean minimum concentration of CBDA was 12.3 ng/mL. These were observed at 16.7 h and 9.3 h after the initial IH feeding respectively. The geometric mean of the terminal half-life (T½) was 15.3 h. A geometric mean residence time of 822.2 h was determined, and the geometric mean AUC_0-∞_ was 13,569.6 h × ng/mL. The mean accumulation index was 1.5 for the 14-day feeding period. No cannabinoids were detected in the plasma of the CNTL cattle.

**Table 1:** Individual animal weights (kg) and doses (mg) of individual cannabinoids administered to each animal in the HEMP group.

	Animal ID								Mean	SD
	1	2	3	4	5	6	7	8		
Body weight, kg	479.5	513.6	465.9	464.1	634.1	420.9	504.5	472.7	494.4	59.0
**Cannabinoid doses, mg/kg**										
Cannabidiolic acid (CBDA)	5.7	5.3	5.9	5.9	4.3	6.5	5.4	5.8	5.6	0.6
Cannabidivarinic acid (CBDVA)	0.005	0.005	0.005	0.005	0.004	0.006	0.005	0.005	0.005	0.00
Tetrahydrocannabinolic acid-A (THCA-A)	0.12	0.11	0.12	0.12	0.09	0.14	0.11	0.12	0.12	0.01
Cannabichromenic acid (CBCA)	0.15	0.14	0.16	0.16	0.11	0.17	0.14	0.15	0.15	0.02
Cannabidiol (CBD)	0.18	0.17	0.19	0.19	0.14	0.21	0.17	0.19	0.18	0.02
9-Tetrahydrocannabinol (9-THC)	0.05	0.05	0.05	0.05	0.04	0.06	0.05	0.05	0.05	0.01
Cannabigerolic acid (CBGA)	0.19	0.18	0.20	0.20	0.15	0.22	0.18	0.20	0.19	0.02
Cannabigerol (CBG)	0.012	0.011	0.012	0.012	0.009	0.014	0.011	0.012	0.01	0.00
Cannabichromene (CBC)	0.07	0.06	0.07	0.07	0.05	0.08	0.06	0.07	0.07	0.01
Cannabinol (CBN)	ND^a^	ND	ND	ND	ND	ND	ND	ND		
Cannabidivarin (CBDV)	ND	ND	ND	ND	ND	ND	ND	ND		

^a^ND Cannabinoids not detected in IH.

**Table 2:** Pharmacokinetic parameters of cannabidiolic acid (CBDA) following feeding industrial hemp for 14 days at a target dose of 5.5 mg/kg (n = 8).

Parameter	Unit	Geometric Mean	Median	Range
λ_z_	1/h	0.05	0.05	0.04–0.06
T1/2	h	15.3	14.8	11.3–19.8
Tmax	h	16.7	24.0	8.0–24.0
Cmax	ng/mL	22.1	22.2	12.4–57.9
AUC_0-∞_	h x ng/mL	13,569.6	13,595.5	8888.6–25,564.7
AUC_0-last_	h x ng/mL	13,508.4	13,542.7	8819.2–25,465.8
AUC extrapolated	%	0.38	0.49	0.15–0.78
MRT_0-∞_	h	822.2	771.5	573.2–1424.8
Accumulation index		1.52	1.49	1.30–1.76

Note: λ_z_, first order rate constant associated with the terminal (log-linear) portion of the kinetic curve; T1/2, terminal half-life; Tmax, the time of maximum observed concentration during the first dosing interval; Cmax, the maximum observed concentration at the first Tmax during the first dosing interval; AUC_0-∞_, area under the concentration curve (AUC) from the dosing time extrapolated to infinity based on the last observed concentration; AUC_0-last_, AUC from the time of dosing to the last measurable concentration; AUC extrapolated, percentage of AUC_0-∞_ due to extrapolation from Tlast to infinity, MRT_0-∞,_ mean residence time extrapolated to infinity; Accumulation index, the ratio of accumulation of a drug under the steady state condition after repeated administration as compared to a single dose.

### Activity monitoring

**Figure 2:**
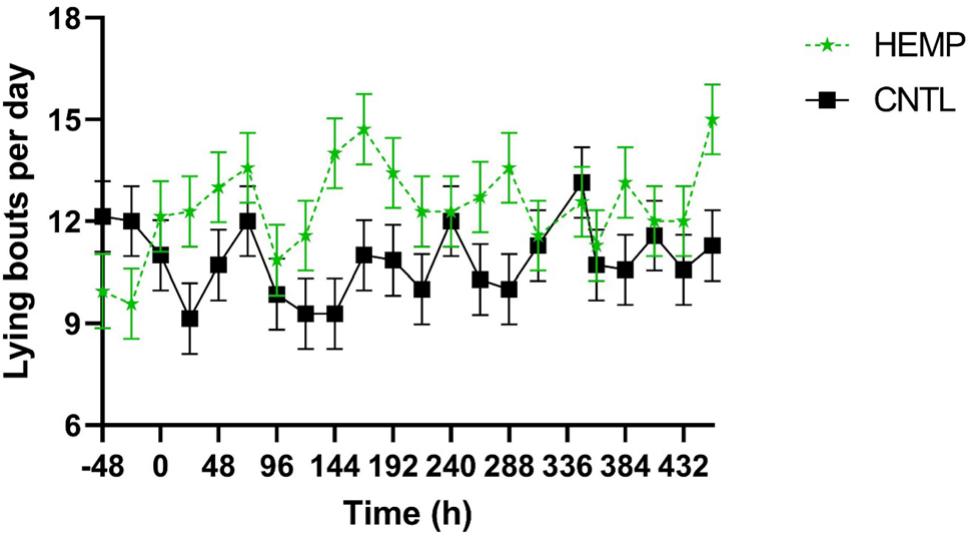
Mean lying bouts for cattle fed 25 g industrial hemp (HEMP; n = 8) for 14 days compared to cattle not fed industrial hemp (CNTL; n = 8).

Activity data are summarized in Table 3. Cattle in the HEMP group recorded a mean 1552 steps per day compared to 1547 steps per day for the CNTL cattle (*P* = 0.97). However, a time effect was observed (*P* < 0.0001) as well as a treatment by time interaction (*P* < 0.0001). Despite no differences in step count, cattle in the HEMP group spent more time lying down compared to the CNTL cattle. Cattle in the HEMP group spent a mean 14.1 h/d (95% CI 13.6–14.6 h/d) lying compared to the 13.4 h/d (95% CI 12.9–138. h/d) for the CNTL cattle (*P* = 0.03). Lying bouts between groups was not different with HEMP having12.4 bouts/d (95% CI 11.0–13.9 bouts/d) compared to CNTL cattle with 10.9 bouts/d (95% CI 9.4–12.2 bouts/d; *P* = 0.12). A treatment by time interaction was observed (Fig. 2) as the HEMP cattle had few lying bouts prior to IH feeding and then increasing bouts/d after IH feeding. Mean motion index in the HEMP cattle [7099.6 (95% CI 6256.8–7942.4)] was lower than CNTL [7222.3 (95% CI 6379.6–8065.1)], but this was not significant (P = 0.83). A significant time effect was seen (P < 0.0001), as well as a treatment by time interaction (P < 0.0001) for motion index between groups. HEMP cattle has lower baseline (pre-dosing) motion indexes compared to CNTL, but these differences were not observed following IH feeding.

**Table 3:** Summary of mean (95% confidence interval) accelerometer data over 24 h intervals for cattle fed industrial hemp once a day for 14 days (HEMP) or control cattle (CNTL).

Parameter	HEMP (n = 8)	CNTL (n = 8)	P- value		
			Treatment	Time	Treatment x time
Steps	1552.2 (1367.9–1736.5)	1547.2 (1362.9–1731.5)	0.97	< 0.0001	< 0.0001
Standing time, h/d	9.9 (9.4–10.4)	10.6 (10.2–11.1)	0.03	< 0.0001	< 0.0001
Lying time, h/d	14.1 (13.6–14.6)	13.4 (12.9–13.8)	0.03	< 0.0001	< 0.0001
Lying bouts, #/d	12.4 (11.0–13.9)	10.9 (9.4–12.3)	0.12	0.01	0.0003
Motion index	7099.6 (6256.8–7942.4)	7222.3 (6379.6–8065.1)	0.83	< 0.0001	< 0.0001

### Blood inflammatory biomarkers

All results are presented as mean ± SEM unless otherwise noted. Inflammatory and stress biomarker data are summarized in Table 4.

**Table 4:** Summary of mean (95% confidence interval) cortisol and prostaglandin E_2_ (PGE_2_) concentrations for cattle fed industrial hemp once a day for 14 days (HEMP) or control cattle (CNTL).

Parameter	HEMP (n = 8)	CNTL (n = 8)	P- value		
			Treatment	Time	Treatment x time
Cortisol, ng/mL	1.59 (− 0.22–3.40)	5.97 (4.43–7.50)	0.001	0.58	0.12
PGE_2_, pg/mL	42.6 (38.0–47.2)	48.2 (43.6–52.8)	0.09	0.053	0.88
PGE_2_ percent change from baseline	− 8.8% (− 20.7–3.0%)	10.2% (− 1.7–22.1%)	0.03	0.08	0.51
Haptoglobin, mg/dL	8.66 (8.14–9.17)	8.63 (8.11–9.17)	0.93	0.61	0.94
Serum Amyloid A, µg/mL	12.65 (7.80–20.53)	8.91 (5.48–14.46)	0.29	0.0007	0.0008

#### Cortisol

Cattle in the HEMP group had lower mean cortisol concentrations than the CNTL cattle (*P* = 0.0013). These differences were most notable on day 14 and day 19. On day 14, HEMP cattle had mean cortisol concentrations of 0.76 ± 0.32 ng/mL compared to 6.57 ± 0.93 ng/mL (*P* = 0.002) in the CNTL group. Similarly on day 19 the HEMP cattle had mean cortisol concentrations of 2.75 ± 1.14 ng/mL and the CNTL had mean concentrations of 7.90 ± 1.28 ng/mL (*P* = 0.004).

#### Ex vivo* prostaglandin E*_*2*_

There tended to be treatment differences observed between treatment groups for PGE_2_ concentrations (*P* = 0.09). Cattle in the HEMP group had mean PGE_2_ concentrations of 46.5 ± 3.9 pg/mL, 42.4 ± 5.2 pg/mL, 46.0 ± 5.0 pg/mL, and 33.2 ± 2.0 pg/mL for the baseline, day 7, day 14, and day 19 time points. Cattle in the CNTL group had mean PGE_2_ concentrations of 48.8 ± 8.1 pg/mL, 51.0 ± 9.5 pg/mL, 53.5 ± 2.7 pg/mL, and 42.0 ± 2.8 pg/mL for the baseline, day 7, day 14, and day 19 time points.

There were differences in the percent change of PGE_2_ from baseline between groups (*P* = 0.03). Cattle in the HEMP group had mean percent changes in baseline of − 6.25 ± 13.2%, − 0.3 ± 9.0% and − 24.4 ± 8.8% for days 7, 14, and 19 respectively. Cattle in the CNTL group had mean percent changes in baseline of 11.9 ± 16.7%, 24.6 ± 14.3% and − 0.18 ± 14.3% for days 7, 14, and 19.

#### Haptoglobin

No differences in haptoglobin were observed between HEMP and CNTL cattle. Mean haptoglobin concentrations were 8.66 ± 0.14 mg/dL and 8.63 ± 0.19 mg/dL for the HEMP and CNTL cattle respectively (*P* = 0.93). Haptoglobin concentrations did not change over time (*P* = 0.61).

#### Serum amyloid A (SAA)

A significant treatment by time interaction was observed. Cattle in the HEMP group had elevated SAA concentrations compared to the CNTL group at 7 d (53.0 ± 1.4 µg/mL vs. 8.2 ± 1.4 µg/mL (*P* = 0.0065)). Mean SAA concentrations were not different between groups at other time points.

#### Hepatic enzyme concentrations

Mean hepatic enzyme concentrations are presented in Table 5. All samples for total protein, albumin, globulin, aspartate transaminase (AST), gamma glutamyltrasferase (GGT) were within the reference intervals for the reporting diagnostic laboratory (KSVDL). Animals in both groups had elevated alkaline phosphatase (ALP) at all time points. There were no significant treatment effects (*P* = 0.87) or treatment by time interactions (*P* = 0.44) observed for ALP between groups. Significant differences for AST were observed with mean CNTL levels of 80.4 ± 3.1 U/L compared to HEMP at 69.8 ± 3.1 U/L (*P* = 0.03). A treatment difference for GGT was observed. The mean CNTL GGT levels (11.7 ± 0.8 U/L) were lower than the GGT levels of the HEMP cattle (14.1 ± 0.8 U/L; *P* = 0.05).

**Table 5:** Summary of mean (95% Confidence intervals) hepatic specific serum blood chemistry profiles prior to industrial hemp feeding and at 7, 14, and 19 days post feeding for cattle fed industrial hemp once a day for 14 days (HEMP) or control cattle (CNTL).

Parameter (Ref range)	HEMP n = 8				CNTL n = 8				*P*-value Treatment
	0	7	14	19	0	7	14	19	
Total protein (6.0–9.0 g/dL)	6.3 (6.1–6.5)	6.7 (6.5–6.9)	6.7 (6.5–6.9)	6.6 (6.4–6.8)	6.4 (6.2–6.7)	6.6 (6.4–6.8)	6.7 (6.5–6.9)	6.8 (6.6–7.0)	0.60
Albumin (3.1–4.3 g/dL)	3.4 (3.3–3.5)	3.5 (3.3–3.6)	3.6 (3.4–3.7)	3.6 (3.4–3.7)	3.3 (3.1–3.4)	3.4 (3.3–3.5)	3.5 (3.4–3.6)	3.5 (3.4–3.5)	0.33
Globulin, g/dL (n/a^1^)	2.9 (2.7–3.1)	3.2 (3.0- 3.4)	3.1 (2.9–3.3)	3.1 (2.9–3.3)	3.2 (3.0–3.4)	3.2 (3.0–3.3)	3.3 (3.1–3.4)	3.3 (3.1–3.4)	0.27
AST^2^ (53–156 U/L)	71.5^a,b^ (64.5–78.5)	68.1^b^ (61.1–75.1)	70.5^a,b^ (63.5–77.5)	68.9^b^ (61.9–75.9)	82.8^c^ (75.8–89.7)	79.9^a,c^ (72.9–86.9)	79.0^a,c^ (72.0–86.0)	80.0^a,c^ (73.0–87.0)	0.03
ALP (21–81 U/L)	189.6 (145.1–234.1)	178.3 (133.7–222.8)	163.3 (118.7–207.8)	165.9 (121.4–210.4)	180.1 (135.6–224.6)	188.0 (143.5–232.5)	175.3 (130.7–219.8)	173.0 (128.5–217.5)	0.87
GGT (10–39 U/L)	14.4^a,b^ (12.4–16.4)	14.9^a^ (12.9–16.9)	14.0^a,b^ (12.0–16.0)	13.3^a–c^ (11.3–15.2)	11.0^c^ (9.0–13.0)	11.6^b,c^ (9.6–13.6)	12.1^a–c^ (10.1–14.1)	12.0^b,c^ (10.0–14.0)	0.05
SDH (6.1–18.4 U/L)	15.7^a,b^ (13.6–17.7)	14.3^a^ (12.2–16.3)	15.1^a,b^ (13.0–17.1)	13.6^a^ (11.5–15.6)	15.5^a,b^ (13.4–17.5)	17.6^b^ (15.6–19.7)	16.3^a,b^ (14.3–18.4)	17.3^b^ (15.3–19.4)	0.08

^a,b^^,c^ Different letter within rows are significantly different (*P* < 0.05).

^d^n/a; calculated value. No laboratory reference given.

^e^*AST* aspartate transaminase, *ALP* alkaline phosphatase, *GGT* gamma glutamyltrasferase, *SDH* sorbitol dehydrogenase.

Sorbitol dehydrogenase (SDH) levels tended to be higher in CNTL cattle (16.7 ± 0.7 U/L) compared to HEMP cattle (14.7 ± 0.7 U/l) (*P* = 0.08). There was a significant treatment by time interaction observed where mean SDH levels in the CNTL cattle increased from15.5 U/L prior to study commencement to 17.3 U/L on day 19. The HEMP cattle mean SDH levels decreased from 15.7 U/L prior to IH feeding to 13.6 U/L on day 19 (*P* = 0.04). All mean levels were below the upper range of the reference interval for the reporting laboratory. However, there were 11 sample above the upper end of the laboratory’s reference range of 18.4 U/L.

## Discussion

The current study was set to investigate the plasma concentrations of cannabinoids in cattle following daily dosing; and the effects of cannabinoids on activity and blood inflammatory and stress biomarkers. This is the first report of repeated IH administration to cattle, and subsequent impact on activity and selected blood parameters. The data here shows cannabinoid administration by daily feeding of IH impacts behavior and the stress potential response in cattle. These findings are important as IH may present a viable way to mitigate stressful experiences such as transportation and regrouping of cattle.

Cattle in the HEMP group were individually hand fed IH daily as part of their grain ration to ensure complete intake. At each feeding, cattle were monitored by researchers to ensure the complete IH dose was consumed. To prevent sorting, the IH was chopped for better mixing with the grain, and molasses was applied just prior to feeding. The amount of IH fed was based on the mean body weight of the animals in the HEMP group obtained on the day of randomization and activity monitor attachment. All cattle received the same amount of IH to replicate a group feeding rate.

No cannabinoids were detected in any of the CNTL cattle samples. The cannabinoids detected in plasma of HEMP cattle of this study were similar to those previously reported with CBDA being the predominate cannabinoid. A similar terminal half-life of 15 h was observed and it is comparable to those previously published of 14 h^5^. The maximum CBDA concentrations (Cmax) following the first dose were lower than those previously reported (22 ng/mL vs 72.7 ng/mL). Time to reach maximum concentrations after the first dose were longer than those published. These differences highlight the need of additional work to better understand the absorption, distribution, metabolism, and excretion of cannabinoids in cattle.

Based on the accumulation ratio of CBDA in this study, there is evidence of drug accumulation^6^. The mean accumulation ratio was 1.52 with a range of 1.30 to 1.76. This indicates concentrations at steady-state were approximately 1.5-times the mean concentrations during the first dosing period (24 h). Further work is needed to determine if this accumulation is clinically relevant. Based on visual inspection of the data, plasma CBDA steady state concentrations were reached at approximately 7 days of IH administration.

The cannabinoids CBDVA, CBGA, and THCA-A were detected in the plasma of all HEMP cattle. The doses for each were 0.005 mg/kg for CBDVA, 0.2 mg/kg for CBGA, and 0.1 mg/kg for THCA-A. Despite being present in a relatively small doses of 0.005 mg/kg in the current study and 0.02 mg/kg in Kleinhenz et al. 2020; CBDVA is found in relatively higher concentrations than other cannabinoids. Cannabidiol (CBD) was detected in 159 of the 288 samples from HEMP cattle, and CBCA was detected in 93 of 288 samples from HEMP cattle. The impact the rumen environment may have on cannabinoids is unclear and deserves further investigation.

Differences in the doses of cannabinoids other than CBDA are due to variation in the cannabinoid content of IH. The variety used in the current study was “Otto II Stout” and the cannabinoid profile was different than the variety used by Kleinhenz et al.^5^. A representative sample was used to analyze the IH for cannabinoid content, but variation within the lot cannot be ruled out. This poses an area of concern if IH were to be approved as an animal feed ingredient.

Cattle in the HEMP group had lower PGE_2_ levels compared to CNTL cattle. Additionally, a decrease from baseline for PGE_2_ was observed for cattle in the HEMP group and compared to the increase in PGE_2_ from baseline for cattle in the CNTL group. These findings suggest a cannabinoid linked reduction in PGE_2_ expression. Data by Takeda et al. demonstrated CBDA has COX-2 inhibitory properties^7^. Additionally, CBDA has been shown to have anti-inflammatory and anti-hyperalgesia effects in rats with carrageenan induce inflammation^8^. It has been suggested that the phenolic ring in CBDA’s structure may mimic salicylic acid^9^. Further investigation is needed to determine if the reduction in PGE_2_ from cannabinoid exposure is clinically relevant in cattle.

There were observed differences in hepatic specific serum biochemistry parameters. Hepatic enzymes were evaluated as evidence suggests cannabinoids are metabolized by liver microsomes^10^. Elevated serum ALP has been reported in dogs administered CBD oil^11^. Interestingly, CBD oil administered to cats did not consistently result in elevated liver enzymes^12^. Although differences in hepatic enzymes were seen between the HEMP and CNTL cattle; most parameters were within the reference range of the laboratory (KSVDL). Thus, these observed differences likely have no biological significance. Alkaline phosphatase (ALP) was elevated for all cattle and is most likely due to contribution of the bone ALP isoform prominent in growing animals.

The acute phase proteins, serum amyloid A and haptoglobin are indicators of inflammation, and were included to investigate if IH feeding resulted in an inflammatory response. Feeding IH caused an increase in SAA concentrations at 7 d in the present study in cattle in the HEMP group. No corresponding increase in haptoglobin was observed in the HEMP group at that time. Although ALP was elevated in all cattle, other hepatic specific enzymes measured (SDH and GGT) were within normal reporting limits for the lab. The impact IH and/or cannabinoids had on the increased SAA is not clear as there is no published veterinary literature on these interactions. Furthermore, the role that IH and cannabinoids may have in mitigating SAA production following an inflammatory or disease event in cattle is not known and deserves further research.

Cortisol concentrations were lower in the HEMP cattle compared to CNTL cattle. This is interesting as both groups were managed identically, but the HEMP group had twice daily venipunctures to obtain blood for cannabinoid concentrations. Synthetic cannabinoid receptor agonists given intravenously provoked an increase in serum cortisol, but hypoalgesia to cutaneous pain and thermal stimuli^13^. Cannabinoids, specifically cannabidiol, have been shown to reduce stress and anxiety in mice^14^. Conversely, CBD did not lower cortisol in dogs following a simulated fireworks model^15^.

Accelerometers have been shown to be an accurate measure of cattle behavior^16^. Raw accelerometer data were condensed to a 24 h period to remove the diurnal effect of cattle’s natural behavior. There were significant differences in the activity of HEMP and CNTL cattle. Most notable were the change in lying time. Cattle in the HEMP group had fewer lying bouts prior to the start of IH feeding and increased lying time after IH feeding started. Lying behavior has been associated with cattle welfare with longer lying time indicating better welfare^17^. A significant treatment by time interaction was observed for motion index. Motion index is a measure of the animal’s overall activity and has been correlated to positive behavior such as running and jumping^18^. Cattle in the HEMP group had lower mean motion indexes during the acclimation period (− 48 h dosing), but an increase was observed following the first dose of IH.

These results, coupled with the cortisol data, suggest cattle fed cannabinoids via IH lower stress biomarkers and improved lying times. Further work is needed to determine if cannabinoids can alter the stress response in cattle during stressful times such as transportation and weaning. The stress biomarkers tested in this study were selected based on their relation to bovine respiratory disease following transportation^19^. Additionally, elevated PGE_2_ and cortisol concentrations have been negatively associated with transportation stress and subsequent disease in the feeding period^20^. The reduction in PGE_2_ and cortisol observed in this study support the potential for IH to reduce transportation stress. Based on the pharmacokinetics of cannabinoids following IH administration, the minimal time from IH feeding to stressful event should be no less than 16 h. This recommendation is based on the reported time to maximum concentrations (Tmax) of 16 h.

## Material and methods

### Ethics statement

This study was performed in accordance with all relevant legislative and regulatory requirements in the United States, the State of Kansas, and AAALAC. All experimental procedures were approved by the Institutional Animal Care and Use Committee at Kansas State University (IACUC# 4421) and are in compliance with the ARRIVE guidelines. Industrial hemp was grown and handled under license of the Kansas Department of Agriculture Industrial Hemp Research Program (licenses numbers: KDA-0621466839 and KDA-0302873296).

### Subjects and housing

The study was conducted in the May of 2021 in northeast Kansas. Sixteen (16) male castrated Holstein cattle weighing (± SD) 447 ± 68 kg were enrolled onto the study. Cattle had been acclimated at the research facility and halter trained prior to study commencement. All cattle were examined and deemed healthy by a veterinarian prior to enrollment. Cattle were group housed in outdoor dirt pens with access to shelter. Pen size per calf exceeded the recommendations set forth in the Guide for the Care and Use of Agricultural Animals in Research and Teaching^21^. Cattle had ad libitum access to grass hay and water via self-filling trough. In addition to the research feed, cattle were fed a custom grain mix at 8:00 and 16:00 h by animal care staff.

### Animal groups

Cattle were randomly assigned by tag number to one of two treatment groups with calf as the experimental unit. Treatment groups were:

1. **HEMP** (n = 8)–cattle fed 25 g IH mixed in 200 g grain once a day for 14 days; or
2. **CNTL** (n = 8)–control cattle fed grain only for 14 days.

### Industrial hemp dosing and cattle sampling

Cattle in the HEMP group were individually fed 25 g IH mixed in 200 g grain placed in rubber feed pans at 07:00 daily for 14 days. The IH was fed at 25 g per animal per day to target a mean dose of CBDA of 5.5 mg/kg. The target dose was based on the administered dose published by Kleinhenz et al.^5^. Daily IH feeding was after the daily blood sample for cattle in the HEMP group.

Cattle were blood sampled at predetermined time points from the jugular or coccygeal veins using a needle and evacuated tubes (Vacutainer, Becton Dickinson, Franklin Lakes, NJ). Whole blood was collected into tubes containing sodium heparin, EDTA, sodium citrate, and no additives. Blood samples from the HEMP cattle were obtained prior to initial IH feeding (day-1) and 8 and 12 h post-initial IH feeding; then every 12 h (prior to the next IH feeding); then (starting 12 h after the last IH feeding) every 24 h for 5 samples or 108 h after the last IH feeding. Cattle in the CNTL group were blood sampled–24 h prior to the initial IH feeding; and on day 7, 14 and 19 post-initial IH feeding. Blood samples, where indicated, were centrifuged at 3,000 *g* for 10 min at 4 °C. Plasma and serum samples were stored at − 80 °C until analyzed.

### Plasma cannabinoid analysis

Plasma cannabinoid concentrations were determined using UPLC-MS methods described by Kleinhenz et al.^5^. All solvents used such as methanol, acetonitrile, isopropanol, formic acid were LC–MS grade. Cannabinoids standards were purchased in individual solutions in methanol (Cerilliant Corporation, Round Rock, TX), including: ( ±)-cis-11-Nor-9-carboxy- Δ 9-tetrahydrocannabinol glucuronide (THC-glu), ( ±)-11-Hydroxy-Δ9-tetrahydrocannabinol (THC-OH), cannabidivarinic acid (CBDVA), cannabidivarin (CBDV), cannabidiol (CBD), cannabidiolic acid (CBDA), Δ9-Tetrahydrocannabinolic acid A (THCA-A), cannabigerolic acid (CBGA), cannabigerol (CBG), Δ9-tetrahydrocannabinol (9-THC), Δ8-tetrahydrocannabinol (8-THC), cannabichromene (CBC), Δ9-tetrahydrocannabivarin (THCV), cannabichromenic Acid (CBCA), cannabinol (CBN), (-)-11-nor-9-Carboxy- Δ 9-tetrahydrocannabinol (THC-acid). Cannabinoid analogs used as internal standards were also purchased in solution in methanol at 100 µg/ml (Cerilliant Corporation, Round Rock, TX): ( ±)-cis-11-Nor-9-carboxy-Δ9-tetrahydrocannabinol glucuronide (THC-glu-d_3_), Cannabidiol-d_3_ (CBD-d_3_), Δ9-Tetrahydrocannabinol-d_3_ (9-THC-d3), ( ±)-11-nor-9-Carboxy-D9-tetrahydrocannabinol-d_9_ (THC-acid-d_9_), ( ±)-11-Hydroxy-Δ9-tetrahydrocannabinol-d_3_ (THC-OH-d_3_), Cannabichromene-d_9_ (CBC-d_9_). All cannabinoids standards were kept in the freezer at − 20 °C.

On the day of analysis, plasma samples were allowed to thaw at room temperature for 2 h. Plasma (samples, quality controls or negative control plasma) were mixed with the internal standard mixture at 200 ng/mL (not added to the negative control sample) and acetonitrile with 0.1% formic acid to precipitate the proteins. The mixture was vortexed for 5 s and centrifuged for 5 min at 7000 *g*. The supernatant was then diluted with ultra-pure 18 Ω water before clean-up. The sample was loaded on a solid phase extraction plate using positive pressure nitrogen. Each well was washed twice with 0.25 mL of a mixture of methanol–water (25:75). The compounds were eluted with two-25 µL aliquots of acetonitrile-methanol (90:10) and diluted with 50 µL of water before analysis.

Cannabinoid analysis was performed using an Acquity H class UPLC and a TQ-S triple quadrupole mass spectrometer (Waters Corp., Milford, MA). The chromatographic separation was performed with a UPLC column (Eclipse Plus C18, Agilent Technologies, Santa Clara, CA) 100 × 2.1 mm, 1.8 µ, heated at 55 °C. The flow rate was set at 0.5 mL/min, the mobile phase consisted of a gradient of acetonitrile (B) and water containing 0.1% formic acid (A) as follow: 0 min: 60%B, 6.50 min: 86% B, 7.50–9 min: 100%B, 9.01 m in12 min: 60%B. The total run time was 12 min. The injection volume was 5 µL. The data acquisition was performed by electrospray ionization in positive and negative mode using multiple reaction monitoring. Linear regression with a weighing factor of 1/X was used and accepted if the coefficient of correlation R^2^ was > 0.99. Calibration curves were linear from 0.1 to 100 ng/mL for all cannabinoids. The lower limit of detection, lower limit of quantification, intra-day precisions, inter-day precisions, and inter-day accuracies for each cannabinoid analyte are summarized in Table 6.

**Table 6:** Lower level of detection (LOD), lower limit of quantification (LOQ), intra-day precisions, inter-day precisions, and inter-day accuracies for each cannabinoid analyte analyzed following oral administration of industrial hemp.

Cannabinoid analytes	LOD^a^	LOQ^b^	Intra-day precision (n = 3)			Inter-day precision (n = 6)			Inter-day accuracy (n = 6)		
			1.75	47.5	95.0	1.75	47.5	95.0	1.75	47.5	95.0
Tetrahydocannabinol-glucuronide (THC-glc)	0.2	0.5	1.5	3.0	4.5	10.5	8.6	7.2	106	105	106
Cannabidivarinic acid (CBDVA)	0.2	1.0	12.3	7.8	14.7	32.5	33.8	33.6	121	115	119
Hydroxy-tetrahydrocannabinol (THC-OH)	0.2	0.25	2.2	6.1	3.7	7.8	5.7	5.8	110	103	103
Tetrahydrocannabinolic-acid (THC-acid)	0.2	0.5	5.7	2.4	4.0	4.2	3.5	5.2	112	103	103
Cannabidivarin (CBDV)	0.2	0.25	4.3	2.6	4.0	6.8	8.5	6.6	105	99	99
Cannabichromenic acid (CBCA)	1.0	2.5	16.7	9.2	6.8	24.7	29.2	24.3	105	99	99
Tetrahydrocannabivarin (THCV)	0.2	0.25	4.3	3.4	3.0	4.5	5.8	6.0	105	110	99
Cannabigerol (CBG)	0.2	0.25	4.8	3.0	2.5	8.3	10.5	12.0	107	100	95
Cannabidiol (CBD)	0.2	0.5	8.4	3.5	4.2	6.9	4.6	6.0	113	103	101
Cannabinol (CBN)	0.2	0.5	1.5	3.4	3.6	5.8	5.2	5.6	105	97	95
9-Tetrahydrocannabinol (9-THC)	0.2	0.5	4.0	3.1	4.1	4.0	3.3	4.0	110	104	103
8-Tetrahydrocannabinol (8-THC)	0.2	0.5	6.3	4.0	3.6	5.3	5.5	5.3	104	100	101
Cannabichromene (CBC)	0.2	0.5	3.8	2.4	2.1	3.3	3.6	3.9	112	103	103
Tetrahydrocannabinolic acid-A (THCA-A)	1.0	2.5	8.5	3.7	8.0	18.2	5.3	8.7	100	89	95
Cannabichromenic acid (CBCA)	1.0	2.5	3.6	4.7	9.3	20.3	7.3	10.9	113	100	108

^a^*LOD* lower limit of detection.

^b^*LOQ* lower limit of quantification.

### Cannabinoid pharmacokinetic analysis

Pharmacokinetic analysis for repeated dosing was performed on each HEMP animal to determine the pharmacokinetics of CBDA using non-compartmental methods using computer software (Phoenix 8.2, Certara, Inc., Princeton, NJ, USA) using methods similar to Kleinhenz et al.^22^. Relevant pharmacokinetic parameters are defined and reported in Table 2.

### Activity monitoring

Cattle in both groups had daily activity monitored via commercially available accelerometers (IceQube, IceRobotics Ltd, South Queensferry, Edinburgh, Scotland UK). Accelerometers were placed on the left rear legs 96 h prior to the first hemp feeding. Accelerometer ID was paired with animal ID prior to placement and at the time of removal 5 days after the final IH feeding (day 19). Steps, time standing up, time lying down and lying bouts, and motion index data was collected via accelerometers at 15 min intervals. Motion index is a measure of the animal’s activity and is correlated to the animals 3-D acceleration including running and jumping^18^. Raw data were downloaded using a RFID reader and computer software (IceManager 2014, IceRobotics Ltd, South Queensferry, Edinburgh, Scotland UK).

Raw data analysis was performed using methods previously described by our lab^23^. Steps, lying bouts and motion index were summated into 24-h increments starting at 07:00 am on day-3 (72 h prior to first feeding) and ending at 7:00 am on day 19. Standing time and lying time were analyzed together due to their interrelation and summed on a 24 h increment to account for the recording method of the accelerometer.

### Blood inflammatory biomarkers

#### Cortisol

Serum cortisol was determined using methods adapted by Kleinhenz et al.^24^. Serum was collected from HEMP and CNTL cattle at − 24 h, and 7, 14, and 19 d post-IH feeding for cortisol determination using a commercially available radioimmunoassay (RIA) kit (MP Biomedicals, Irvine, CA, USA) following manufacturer specifications with minor modifications. The standard curve was extended to include 1 and 3 ng/mL by diluting the 10 and 30 ng/mL manufacturer-supplied standards 1:10 respectively for a detection range of 1 to 300 ng/mL. A low (25 ng/mL) and high (150 ng/mL) quality control (QC) were ran at the beginning and end of each set to determine inter-assay variability. Standard curves were plotted as a 4-parameter logistic curve with an R^2^ of 0.998.

#### Ex vivo* prostaglandin E*_*2*_

Ex vivo PGE_2_ concentration were determined using methods describe by our lab^25^. Briefly, whole blood samples were collected from each animal − 24 h prior to initial IH feeding, and 7, 14, and 19 d post-IH feeding. At each collection, whole blood (1 mL) samples were spiked with 10 µg lipopolysaccharide from *Escherichia coli* 0111:B4 (Sigma-Aldrich, MO, USA). Samples were incubated for 24 h at 37 °C, centrifuged and the plasma pipetted into individual cryovials and stored at − 80 C until analyzed. After thawing, plasma proteins were precipitated using methanol, centrifuged at 3,000 *g* for 10 min and PGE_2_ concentration of the supernatant determined using a commercially available ELISA kit (Cayman Chemicals, MI, USA). The coefficient of variation for intra-assay variability was 10.7% and interassay variability was calculated as 10.8%.

#### Haptoglobin and hepatic enzyme concentrations

Serum samples collected prior to hemp feeding and at 7, 14, and 19 d post-IH feeding were submitted to the Kansas State Veterinary Diagnostic Laboratory for biochemical analysis by photometric methods (Cobas 501, Roche Diagnostics, Indianapolis, IN, USA).

### Statistical analysis

Cortisol concentrations were log transformed for normality prior to analysis. Statistical analysis was performed using computer software (JMP 15.0, SAS Inst. Inc., Cary, NC). Responses were analyzed using a mixed model with calf as the experimental unit. Treatment was assigned as the random effect; and time and treatment by time interaction were considered as fixed effects. Baseline PGE_2_ concentrations were included as a fixed effect when analyzing the PGE_2_ percent change from baseline. Post hoc tests were conducted using Tukey–Kramer adjustment. Statistical significance was set a *P* < 0.05 a priori.

## Data Availability

All data generated or analyzed for this study are included in this article.
